# Longitudinal association between myopia and parental myopia and outdoor time among students in Wenzhou: a 2.5-year longitudinal cohort study

**DOI:** 10.1186/s12886-020-01763-9

**Published:** 2021-01-06

**Authors:** Dandan Jiang, Haishuang Lin, Chunchun Li, Linjie Liu, Haishao Xiao, Yaoyao Lin, Xiaoqiong Huang, Yanyan Chen

**Affiliations:** 1 The Eye Hospital of Wenzhou Medical University, 270 West Xueyuan Road, Wenzhou, Zhejiang, 325027 China; 2 School of Ophthalmology and Optometry, Wenzhou Medical University, 82 West Xueyuan Road, Wenzhou, Zhejiang, 325027 China

**Keywords:** Children myopia, Parental myopia, Outdoor time, Refractive error, Axial length

## Abstract

**Background:**

To evaluate the impact of parental myopia and outdoor time on myopia among students in Wenzhou.

**Methods:**

We examined 1388 primary students from first grade to third grade in Wenzhou from September 2012 to March 2015. We performed noncycloplegic refractometry on each student every six months and axial length (AL) measurements every year. At the commencement of our study, children were asked to complete a questionnaire regarding near work activity and outdoor activity, whereas parents were asked to complete a self-administered questionnaire regarding their background circumstances and their history of myopia.

**Results:**

A total of 1294 students (93.2%) returned for follow-up examinations. Children with initial and final no myopia spent more time on outdoor activities than those with new onset myopia (1.92 vs. 1.81 h/d, *p* = 0.022), and elongation of AL in children with a high level (> 2.5 h/day) of outdoor time (0.22 ± 0.13 mm/Y) was less than those with a low level (≤ 1.5 h/day) of outdoor time (0.24 ± 0.14 mm/Y, *p* = 0.045). The proportion of rapid myopia progression (≤-0.5D/Y) was 16.7%, 20.2% and 31.5% among the children with no myopic parent, one myopic parent and two myopic parents, respectively (X^2^ = 28.076, *p* < 0.001), and the elongation of AL in children among different numbers of myopic parents was significantly different (*p* < 0.001). A high level of outdoor time was a protective factor for children with one myopic parent (HR 0.49, 95% CI 0.27–0.88; *p* = 0.018).

**Conclusions:**

In this sample, parental myopia and outdoor time were associated with myopia in children. A high level of outdoor time was a protective factor for children with one myopic parent.

**Supplementary Information:**

The online version contains supplementary material available at 10.1186/s12886-020-01763-9.

## Background

Myopia is a severe public health problem, especially among Asian students [1]. In China, the prevalence of myopia among junior school students increased from 55.95% in 2005 to 65.48% in 2015 [2], while it increased from 79.5% in 2001 to 87.7% in 2015 among senior high school students [3]. This rapid increase in the prevalence of myopia among the young generation may lead to myopia becoming one of the main blinding causes in the future because high myopia is associated with myopic maculopathy [4] and an increase in glaucomatous optic neuropathy prevalence [5]. Although the causes of myopia remain unclear, numerous studies have suggested its development is caused by environmental and genetic factors [6, 7].

Several myopia-related environmental factors, such as prolonged near work, lack of outdoor exposure and inappropriate reading posture, have been reported [8]. A number of studies suggest that near work and outdoor exposure are significantly associated with children’s myopia problem [9–13]. Near work-related behaviours were related to more myopic spherical equivalent refraction (SER) and longer axial length (AL) [10]. The time spent outdoors and near work activities had an efficient impact on the development of myopia in school children [1]. Epidemiological studies [14–17] reported that parental myopia was a risk factor for children’s myopia, and the risk of developing myopia in children was increased with the number of myopic parents. A meta-analysis [18] of the association between myopia in parents and their child’s risk of developing myopia also demonstrated this relationship. Parental myopia [19–22] was associated with refractive status and AL in both Asian children and European children. Children with myopic parents exhibited an increased prevalence of myopia, more myopic SER and longer AL. Furthermore, Asian children with parental myopia exhibited more myopic SER and longer AL than those children in European children.

Since parents and their children have similar family environments and behavioural habits, parental myopia should not be simply classified as environmental or genetic factors [23]. Saw’s study [24] showed that children with two myopic parents spent reading much more time compared with those with no myopic parent. Thus, modification of the risk of outdoor activities time and near work by parental myopia should be revealed.

The purpose of this study was to evaluate the interactive efficacy of parental myopia, near work and outdoor activity time on myopia onset and progression in Chinese children.

## Methods

### Study design

This study was a school-based longitudinal cohort study. Three primary schools were selected in Wenzhou, China with cluster sampling based on similar socioeconomic status and campus culture. Grade 1–3 children were investigated at baseline and were followed for 2.5 years.

A total of 1388 grade 1–3 students were included in the study, excluding objects with ocular inflammation, trauma, dysgnosia and ill-matching. A total of 1294 students and their parents completed the eye examinations and questionnaire survey at the beginning of every semester from September 2012 to March 2015.

### Questionnaires

Students and their parents completed the self-administered questionnaire at the beginning of the study. The questionnaire for children included basic questions about gender, grade and near work activity (questions on homework, reading, video-game playing and computer use) and outdoor activity. The questionnaire was distributed by taking class as a unit before the eye examinations. The children needed to complete the questionnaires after we gave them the questionnaire and told them how to complete it. Then, the questionnaires were returned it at once.

Parents completed the questionnaire that included basic questions about age, family income, education level, occupation and refractive status. Parents need to answer the following questions: “Did child’s father (or mother) have the following vision problems?” (Myopia/Hyperopia/Astigmatism/NO), “What’s the most myopic or hyperopic refractive of the two eyes?” (<− 6.0D/− 6.0 to − 3.0D/> − 3.0D). At the first time of investigation, children took the questionnaires for their parents and returned it the day after tomorrow. The investigators checked and audited all the questionnaires to increase their validity.

### Examinations

Before the start of the study, every member of the research team was trained, including 2 senior ophthalmologists, 4 experienced ophthalmic nurses and 3 postgraduates. All children included in the study were examined for visual acuity, noncycloplegic refraction and optical biometers. All instruments were checked and adjusted before the examination.

Visual acuity and noncycloplegic refraction were examined by experienced senior ophthalmologists from each term. The examination process began with testing visual acuity at 5 m using a standard logMAR chart. Visual acuity was tested with and without refractive correction for those wearing spectacles. An autorefractor (TOPCON-RM8900) was used to measure noncycloplegic refraction. Each eye of each student was measured without cycloplegia at least thrice, and three repeated measurements were averaged for analysis.

AL, corneal radius of curvature and anterior chamber depth were performed with an optical biometer (Carl Zeiss Meditec AG, 07740 Jena, Germany). We used the average of five valid AL measurements for analysis.

### Definition and statistical analysis

Statistical analysis was performed using SPSS 20.0. SER = sphere + 1/2 cylinder. Myopia was defined by an SE of − 1.0 diopters (D) or more myopic. Parental myopia collected from the questionnaire was classified by the number of myopic parents (no, one and two). The average daily time spent near work and outdoor activities was calculated using the formula: [(hours spent on a weekday) *5+ (hours spent on a weekend day) *2]/7. Outdoor time was divided into three groups as low: ≤ 1.5 h/day (25th percentile), moderate: 1.5–2.5 h/d (50th percentile), and high > 2.5 h/day (75th percentile) for analysis. Chi-square and T-tests were used to compare near work time, outdoor time and proportion of children with different number of myopic parents between children with initial and final no myopic and new set myopia. The comparison of myopia progression and elongation of AL between different numbers of myopic parents by Chi-square test and ANOVA. Kaplan-Meier failure curves to display event rates. Hazard ratios (HRs) with 95% CIs were estimated using the Cox proportional hazards model between children with different levels of time spent on outdoor activities and the number of myopic parents. We used the Cox proportional hazards model for odds of new onset myopia in children to assess the interaction of the number of myopic parents and outdoor activity. All *p*-values were 2-sided and considered statistically significant when less than 0.05.

## Results

A total of 1388 children were enrolled in this study, and 1294 (93.2%) completed eye examinations and self-administered questionnaires six times over a 2.5-year period. Socio-demographic characteristics were compared between those children who was myopia and those was no-myopia (Table 1). No significant difference in the amount of time spent on near work activities and outdoor activities between children who was myopia and those was no-myopia(*p* > 0.05). The proportion of myopic parents was significant different between children who was myopia and those was no-myopia(*p* < 0.001).

**Table 1:** Baseline Characteristics of primary school children

Characteristic	All	Myopia	No-Myopia	*P* value ^*^
*n*	1294	184	1110	/
Age, mean ± SD, year	7.29 ± 0.91	7.59 ± 0.83	7.24 ± 0.91	< 0.001
Sex, *n*(%)	606 (46.8%)	78 (42.4%)	528 (47.6%)	0.192
BMI, mean ± SD, kg/m^2^	16.42 ± 2.33	16.75 ± 2.65	16.37 ± 2.27	0.066
SER, mean ± SD, D	−0.25 ± 0.94	−1.97 ± 0.95	0.03 ± 0.56	< 0.001
AL, mean ± SD, mm	23.05 ± 0.84	23.83 ± 0.98	22.92 ± 0.74	< 0.001
CRC, mean ± SD,,mm	7.81 ± 0.25	7.76 ± 0.24	7.82 ± 0.26	0.006
AL/CRC ratio, mean ± SD	2.95 ± 0.09	3.07 ± 0.10	2.93 ± 0.07	< 0.001
Nearwork time, mean ± SD, h/d	3.25 ± 0.92	3.28 ± 0.90	3.24 ± 0.92	0.654
Outdoor time, mean ± SD, h/d	1.88 ± 0.74	1.85 ± 0.71	1.89 ± 0.75	0.462
Number of myopic parents, *n*(%)				
0	263 (20.3%)	25 (13.6%)	238 (21.4%)	< 0.001
1	489 (37.8%)	51 (27.7%)	438 (39.5%)	
2	542 (41.9%)	108 (58.7%)	434 (39.1%)	

* Chi square test was used in classification variable comparisons, independent t-test was used in continuous variable comparisons

**Fig. 1:**
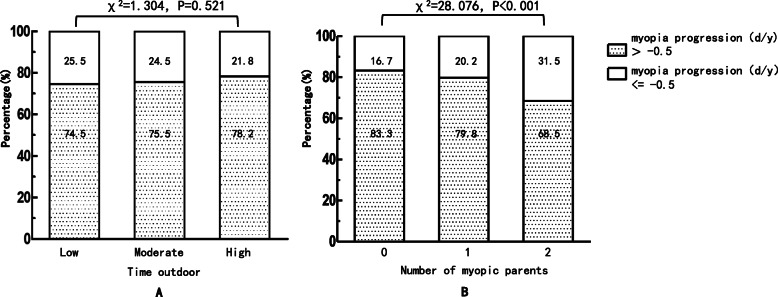
Percentage of myopia progression of all children within different outdoors time **a** and number of myopic parents **b**, respectively

**Fig. 2:**
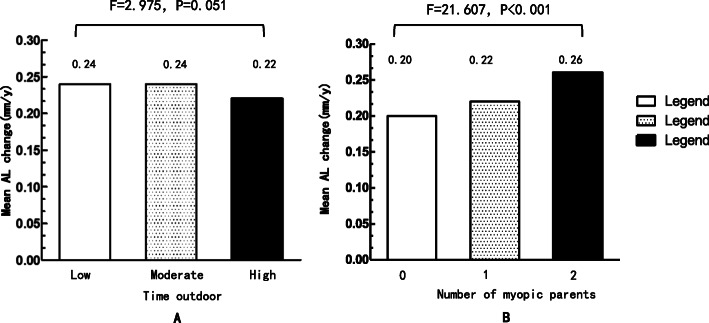
Mean change in AL of all children within different outdoors time **a** and number of myopic parents **b**, respectively

As shown in Table 2, children with new onset myopia reported similar time spent on near work activities with those who were initial and final not myopic (*p* = 0.708). The time spent on outdoor activities was higher in children with initial and final non-myopia than in children with new onset myopia (1.92 vs. 1.81 h/d, *p* = 0.022). The proportion of rapid myopia progression (≤-0.5 D/Y) decreased with time outdoors with no significant difference (X^2=^1.304, *p* = 0.521; Fig. 1a). As shown in Fig. 2a, children with a low level of outdoor time did exhibit significantly greater elongation of the AL (0.24 ± 0.14 mm/Y) compared with children with high levels of outdoor time (0.22 ± 0.13 mm/Y, *p* = 0.045).

**Table 2:** Univariate Results for Refractive Error, Time Activities, and Parental history for children myopia change

Variable	Initial and final non-myopia (*n* = 803)	New onset myopia (*n* = 307)	statistics †	*p* value ‡
Baseline SER, mean ± SD, D	0.15 ± 0.55	−0.28 ± 0.42	12.418	< 0.001
Baseline AL, mean ± SD, mm	22.83 ± 0.72	23.15 ± 0.74	−6.629	< 0.001
Baseline AL/CRC ratio, mean ± SD	2.92 ± 0.06	2.98 ± 0.06	−13.992	< 0.001
Mean myopia progression, mean ± SD, D/y	−0.03 ± 0.23	−0.60 ± 0.36	31.459	< 0.001
Mean AL change, mean ± SD, mm/y	0.17 ± 0.08	0.36 ± 0.12	−29.903	< 0.001
Near work time, mean ± SD, h/day	3.25 ± 0.94	3.23 ± 0.87	0.374	0.708
Outdoor time, mean ± SD, h/day	1.92 ± 0.76	1.81 ± 0.71	2.295	0.022
Number of myopic parents				
0	(191)23.8%	(47)15.3%	87.126	< 0.001
1	(336)41.8%	(102)33.2%	125.014	< 0.001
2	(276)34.4%	(158)51.5%	32.083	< 0.001

† Statistics for children with no-myopia at baseline

‡ Chi square test was used in classification variable comparisons, independent t-test was used in continuous variable comparisons

An increased proportion of no or one myopic parent in children with no myopia was noted compared with children with new onset myopia (*p* < 0.01) (Table 2). However, the proportion of children with two myopic parents was lower in non-myopic children compared with children with new onset myopia (Table. 2). As noted in Fig. 1b, during the 2.5-year period, the proportion of rapid myopia progression (≤-0.5 D/Y) was 16.7, 20.2 and 31.5% in children with no myopic parent (*n* = 44), one myopic parent (*n* = 99) and two myopic parents (*n* = 171), respectively (X^2^ = 28.076, *p* < 0.001). A significant elongation of the AL was noted during the follow-up that was associated with an increasing number of myopic parents during this period (F = 21.607, *p* < 0.001) (Fig. 2b).

We also analysed the interaction outcome of myopia onset using the Cox proportional hazard model after adjusting for age, sex, BMI, baseline SER, baseline AL, and near work time. A high level of outdoor time reduced the risk of reaching an endpoint (HR 0.69, 95% CI 0.49–0.96; *p* = 0.027, supplemental Table 1). Two myopic parents were associated with an increased risk of reaching an endpoint (HR 2.01, 95% CI 1.45–2.78; *p* < 0.001, supplemental Table 1). A significant interaction was noted between time outdoors and number of myopic parents (*p* < 0.05). A high level of outdoor time was a protective factor for children with one myopic parent (HR 0.49, 95% CI 0.27–0.88; *p* = 0.018, Table 3).

**Table 3:** Interaction between Time outdoors, and number of myopic parents on myopia onset of primary school children

Baseline Characteristic	Multivariate model		
	Adjusted Hazard Ratio	95% CI	*p*-value
Sex			
Boy	1 [Reference]		
Girl	1.84	1.43–2.38	**< 0.001**
Age	1.15	1.01–1.31	**0.037**
BMI	1.01	0.95–1.06	0.858
Refractive error	0.19	0.14–0.25	**< 0.001**
Axial length	1.60	1.33–1.93	**< 0.001**
Nearwork time			
Low(0–2.5 h/d)	1 [Reference]		
Moderate (2.5–3.5 h/d)	1.10	0.82–1.48	0.533
High(> 3.5 h/d)	1.07	0.78–1.47	0.661
Time outdoors. Moderate vs. low × Number of myopic parents. 1 vs 0	1.16	0.86–1.57	0.323
Time outdoors. High vs. low × Number of myopic parents. 1 vs 0	0.49	0.27–0.88	**0.018**
Time outdoors. Moderate vs. low × Number of myopic parents. 2 vs 0	1.15	0.85–1.57	0.366
Time outdoors. High vs. low × Number of myopic parents. 2 vs 0	1.14	0.76–1.72	0.536

## Discussion

### Environmental factors

Our study reported that myopia in children was significantly related to outdoor activity time. Epidemiological studies [25, 26] found that outdoor activity time in children with myopia was significantly reduced compared with that in non-myopic children. Mutti et al. [14] reported that outdoor activity time was 9.7 h/week in children with no myopia and 7.4 h/week in children with myopia among 3660 eight grade students with a moderate level of outdoor time. Our study showed similar results. Children with initial and final non-myopia spent more time outdoors than children with new-onset myopia (*p* < 0.05). A high level of outdoor time exhibited a 31% (HR 0.69, 95% CI 0.49–0.96; *p* = 0.027) reduced risk in new myopic children compared with a low level of outdoor time. However, myopia progression was not related to different levels of time spent on outdoor activities (*p* = 0.521) in our study. This finding was consistent with a meta-analysis [27], which demonstrated that outdoor time was not effective in slowing myopia progression in children already diagnosed with myopia. In China, children who spent less time outdoors had greater AL elongation during a 4-year follow-up (standardized coefficient beta: − 0.22, *p* < 0.05, [28]. Given the importance of AL in myopia, we also analysed the association between AL and myopia-associated risk factors. We concluded that children with high levels of outdoor time also did not exhibit quicker elongation of AL compared with children with moderate levels (*p* = 0.051), but significantly quicker elongation was noted compared with children with lower levels of outdoor time (*p* = 0.045). This finding indicates that a high level of outdoor time inhibited AL changes in children.

Near work was also a risk factor for myopia in school children [9, 29, 30]. However, conclusions vary from study to study. A Chinese population-based study [31] reported that more time spent indoors studying was associated with an increased prevalence of myopia in primary school children. The Beijing Myopia Progression Study reported no association between near work time and myopia [26]. In our study, near work time was not associated with the incidence of myopia (*p* = 0.708).

### Parental myopia and children myopia

Our study showed that parental myopia was significantly associated with child myopia. Children with new-onset myopia had a higher proportion of two myopic parents than those with initial and final non-myopia (51.5% vs 34.3%). Moreover, our study showed that two myopic parents represented an increased risk in new myopic children (HR 2.01, 95% CI 1.45–2.78; *p* < 0.001) compared with no myopic parent. This trend was similar to previous studies. For example, one large-sample study among Chinese children [22] reported that the prevalence of myopia was 68.2, 88.9 and 83.3% in children with no, one or two myopic parents, respectively. The effect of parental myopia on children also varied based on the number of myopic parents in Australia [21].

In addition, as the number of myopic parents increased, the proportion of myopia progression (≤ − 0.5 D/Y) increased remarkably (*p* < 0.001). The finding that myopia developed faster in children with myopic parents was consistent with some previous studies [32, 33]. One cohort study [32] showed a remarkable relationship between parental myopia and myopia progression in children, and the rate of myopia progression was − 0.60 D/year in children with one or two myopic parents and − 0.42 in children with no myopic parent. M. H. Edwards et al. [34] proposed that children without myopic parents had smaller AL than children with myopic parents. Our study also showed that children with myopic parents had a quicker rate of elongation of AL (*p* < 0.001), especially in children with two myopic parents.

According to the results of the association between myopia progression and AL elongation in children and parental myopia, the conclusion that parental myopia is one of the risk factors for child myopia can be drawn. A number of studies have consistently shown that children with two myopic parents had a higher risk of developing myopia than children with no myopic parent [14, 15, 18, 35].

### Interaction between parental myopia and outdoor activity

Furthermore, the interaction between parental myopia and outdoor time among children’s myopia was significant. Outdoor activity time was significantly associated with myopia in children within myopic parents. Our study results suggested that a high level of outdoor time resulted in a 58% (HR 0.49, 95% CI 0.27–0.89; *p* = 0.019) reduction in the risk of new myopia for children with one myopic parent.

Many researchers have attempted to explore the interaction between parental myopia and environmental factors among children’s myopia. Saw’s study [24] shows that children with two myopic parents spent much more time reading than those with no myopic parent. A longitudinal study [36] of 514 children reported that parental myopia was an important predictor of children’s myopia, and children with two myopic parents were likely to be myopic when less time was spent on outdoor activities (environmental exposure).

In fact, the influence of genetics and environmental factors on myopia should not be analysed separately. Parents and their children have similar family environments and behavioural habits. Family culture and study environment for children were influenced by parents. The prevalence of myopia in sisters and brothers was still significantly related when the environment they lived changed [37].

The conclusion that parental myopia influences children’s myopia more than environmental factors cannot be completely certain in our study. However, we found that a higher level of outdoor time is important for children, especially children with myopic parents. Higher levels of outdoor time can protect children from outset myopia for those with one myopic parent.

### Limitations of the study

There were some important findings in our study, but there were some potential limitations. First, we performed noncycloplegic refractions given the large number of students in our study. Many parents worry about potential side effects, and most children object to eye drops. It is not possible to maintain a 93.2% completion rate in such a study when using cycloplegia. Gwiazda et al. [38] reported in the COMET study that noncycloplegic refractions are only 0.23 diopters more myopic than cycloplegic refractions. In addition, some experts studied the relationship between myopia and outdoor activity using noncycloplegic refraction [39–41]. Accordingly, noncycloplegic refractometry may result in a slightly higher prevalence of myopia. Second, the time spent outdoor activities was estimated by questionnaires, and recall bias may exist. However, every questionnaire was checked by school teachers and experienced ophthalmic nurses according to the course schedule. The validity and accuracy of these data were assured by checking and excluding unbelievable questionnaires. Third, the refractive error of parents was self-reported. We considered that the accuracy of parental self-reported myopia is credible for the high education of the parents (college degree or greater accounting for 70%). Most parents have a correct understanding of their refractions. Moreover, we evaluated parental myopia status instead of parental refractions, which decreased the influence of inaccurate self-reported myopia.

## Conclusions

In conclusion, this study shows a noticeable relationship between child myopia and parental myopia and outdoor time. We found that a high level of outdoor exposure had a remarkable influence on the risk of new myopia for children even with one myopic parent. Therefore, it is suggested that time spent on outdoor activities for children with myopic parents should be increased. Future studies should explore the mechanism of the interaction between genetic and environmental factors among children’s myopia.

## Supplementary Information

**Additional file 1: Supplemental Table 1.** Factors Associated with myopia onset of primary school children by Cox Proportional Hazard Regression Analysis.

**Additional file 2.** The Wenzhou Epidemiology of Refraction Error (WERE) study Questionnaire

## Data Availability

The datasets analysed in this study are available from the corresponding author (Yanyan Chen, wzcyymail@163.com) upon reasonable request.

## Abbreviations

AL: Axial length
SER: Spherical equivalent refraction
HRs: Hazard ratios
BMI: Body mass index
