# Supplementary material for: Longitudinal association between myopia and parental myopia and outdoor time among students in Wenzhou: a 2.5-year longitudinal cohort study
Source: BMC Ophthalmol. 2021 Jan 6;21:11. doi: 10.1186/s12886-020-01763-9 (PMC7789164; doi:10.1186/s12886-020-01763-9)
Supplement: Supplementary file 1 — Additional file 1: Supplemental Table 1. Factors Associated with myopia onset of primary school children by Cox Proportional Hazard Regression Analysis. [file 12886_2020_1763_MOESM1_ESM.docx]

| Supplemental Table 1. Factors Associated with myopia onset of primary school children by Cox Proportional Hazard Regression Analysis | | | |
| --- | --- | --- | --- |
| Baseline Characteristic | Univariate model | | |
|  | Adjusted Hazard Ratio | 95% CI | p-value |
| Sex |  |  |  |
| Boy | 1 [Reference] |  |  |
| Girl | 1.17 | 0.93-1.46 | 0.172 |
| Age | 1.35 | 1.20-1.53 | **<0.001** |
| BMI | 1.03 | 0.99-1.09 | 0.177 |
| Refractive error | 0.18 | 0.14-0.23 | **<0.001** |
| Axial length | 1.73 | 1.48-2.02 | **<0.001** |
| Number of myopic parents |  |  |  |
| 0 | 1 [Reference] |  |  |
| 1 | 1.21 | 0.86-1.71 | 0.273 |
| 2 | 2.01 | 1.45-2.78 | **<0.001** |
| Outdoor time |  |  |  |
| Low(0-1.5h/d) | 1 [Reference] |  |  |
| Moderate (1.5-2.5h/d) | 0.97 | 0.76-1.24 | 0.785 |
| High(>2.5h/d) | 0.69 | 0.49-0.96 | **0.027** |
| Nearwork time |  |  |  |
| Low(0-2.5h/d) | 1 [Reference] |  |  |
| Moderate (2.5-3.5h/d) | 1.11 | 0.83-1.49 | 0.485 |
| High(>3.5h/d) | 0.97 | 0.71-1.32 | 0.826 |
