# Supplementary material for: Longitudinal association between myopia and parental myopia and outdoor time among students in Wenzhou: a 2.5-year longitudinal cohort study
Source: BMC Ophthalmol. 2021 Jan 6;21:11. doi: 10.1186/s12886-020-01763-9 (PMC7789164; doi:10.1186/s12886-020-01763-9)
Supplement: Supplementary file 2 — Additional file 2. The Wenzhou Epidemiology of Refraction Error (WERE) study Questionnaire [file 12886_2020_1763_MOESM2_ESM.docx]

***English Version***

**The Wenzhou Epidemiology of Refraction Error (WERE) study Questionnaire**

1. Child’s name:
2. Child’s Sex:
3. Child’s Grade , Class
4. Child’s Date of birth:
5. Do you currently wear orthokeratology lenses?

□ Yes □No

6. Please tick the activities you do and fill in the number of hours per day you spend doing the activity.

|  | Yes | On weekday  (From Monday to Friday ) | Yes | On weekend / Winter and Summer holiday |
| --- | --- | --- | --- | --- |
| 1. School homework | □ | hrs per day | □ | hrs per day |
| 1. Read for extra-curriculum activities | □ | hrs per day | □ | hrs per day |
| 1. Playing video games | □ | hrs per day | □ | hrs per day |
| 1. Using a computer | □ | hrs per day | □ | hrs per day |
| 1. Outdoor activities (leisure and sports) | □ | hrs per day | □ | hrs per day |

7. Father’s Date of birth:

8. Mother’s Date of birth:

9. Family’s annual income ?

□ Less than fifty thousand yuan

□ Fifty ~one hundred thousand yuan

□ One hundred~one hundred and half thousand yuan

□ More than one hundred and half thousand yuan

10. Father’s education level:

11. Mother’s education level:

12. Father’s occupation:

13. Mother’s occupation:

14. Did child’s father have the following vision problems?

□Myopia □Hyperopia □Astigmatism □NO

What’s the most myopic or hyperopic refractive of the two eyes?

□<-6.0D □-6.0 to -3.0D □>-3.0D

15. Did child’s mother have the following vision problems?

□Myopia □Hyperopia □Astigmatism □NO

What’s the most myopic or hyperopic refractive of the two eyes?

□<-6.0D □-6.0 to -3.0D □>-3.0D
